# The effect of alcohol on surgical skills

**DOI:** 10.1308/003588412X13373405386097

**Published:** 2012-09

**Authors:** Tim Hems

**Affiliations:** Department of Orthopaedic Surgery, The Victoria Infirmary, Glasgow,UK

## Abstract

**Kirby G, Kapoor K, Das-Purkayastha P, Harries M** The effect of alcohol on surgical skills.*Ann R Coll Surg Engl* 2012; **94**: 90–93 doi: 10.1308/003588412X13171221501627

I have severe concerns regarding the content of the above article and its publication in the *Annals*.The authors state that ‘It is hoped that the results will serve as a guide to safer use of alcohol among surgeons during their time on call’. The implication therefore appears to be that it might be acceptable for a surgeon on call to consume some alcohol and that there is variability in attitudes to alcohol consumption during on-call shifts. This is not an acceptable position for the Royal College of Surgeons to be associated with.

For reasons that go beyond the effect on surgical skills, alcohol should not be consumed by surgeons on call. The only situation in which, on rare occasions, there may be some justification for attending a hospital having consumed some alcohol, is when a surgeon is contacted when not on call.

